# Differentiation of the Nutritional Risk of Polish Elderly People According to Selected Demographic Characteristics and Declared Socioeconomic Status

**DOI:** 10.3390/nu14081582

**Published:** 2022-04-11

**Authors:** Robert Gajda, Ewa Raczkowska, Joanna Wyka, Edyta Suliga, Kamila Sobaś

**Affiliations:** 1 Department of Human Nutrition, Faculty of Biotechnology and Food Science, Wrocław University of Environmental and Life Sciences, 51-630 Wrocław, Poland; robert.gajda@upwr.edu.pl (R.G.); joanna.wyka@upwr.edu.pl (J.W.); 2 Institute of Health Sciences, Medical College, Jan Kochanowski University, 25-516 Kielce, Poland; edyta.suliga@ujk.edu.pl (E.S.); kamila.sobas@ujk.edu.pl (K.S.)

**Keywords:** nutritional risk, elderly, gender, age, socioeconomic status

## Abstract

Nutritional risk screening in older people can help to not only identify health risks but also to treat them effectively. The aim of this work was to assess the relationship between the demographic characteristics (age, gender and place of residence) and socioeconomic status of older people in the community and nutritional risk. The Seniors in the Community: Risk Evaluation for Eating and Nutrition (SCREEN-14) questionnaire was used to evaluate the nutritional risk. The study was conducted in 417 people (312 women and 105 men) between 60 and 95 years old (70.8 ± 6.73 years). Multivariate correspondence analysis (MCA) was used to determine the relationships between the categories of variables describing the level of nutritional risk, demographic characteristics and the value of the socioeconomic status (SES) index. To assess the relationship between identified nutritional risks, demographics and SES index variables, we used logistic regression analysis. Based on these studies, nutritional risk factors for older people in Poland were identified. It has been shown that larger cities and low socioeconomic status are closely linked to higher nutritional risk. At the same time, age and gender were not significant factors influencing nutritional risk. Identifying the factors that increase the nutritional risk of older people can help to improve their quality of life.

## 1. Introduction

Aging is closely related to deterioration in physical and cognitive performance. Therefore, adequate nutrient intake is very important in the elderly [1,2]. Unfortunately, many nutritional errors are observed in this group of people. The most frequently described are the montotonicity of meals; low supply of vegetables and fruits, dairy products, cereal products, fish and water; excessive consumption of sweet and salty snack products, meat and its products, fats and foods with high energy density; and low nutritional density [3]. Consequently, inadequate nutrition can lead to various dysfunctions, such as decreased immunity, brittleness and non-communicable diseases (NCDs). Not only malnutrition but also overweight and obesity significantly increase the risk of these dysfunctions [4,5].

Screening for nutritional risk among the elderly is still of interest to researchers [6,7,8]. This type of research may contribute to the identification of health threats, their effective treatment and be part of the provision of services [8,9]. Despite the consensus around defining malnutrition, there is still no distinction between the definitions of malnutrition risk and nutritional risk [10]. While malnutrition risk refers to individuals characterized by indicators of malnutrition (e.g., very low food intake, weight loss and decreased functional capacity), nutritional risk is a more general term and refers to conditions and factors that are associated with decreased food intake, both in terms of quantity and quality of intake. If these conditions and factors are not eliminated, they can lead to malnutrition over time. Initially, screening tools were concerned with assessing the risk of malnutrition among people in clinical settings, but such tools are now being designed to assess nutritional risk, for different population groups, including those living in local communities [11]. Nutritional risk is a more prevalent occupation than malnutrition risk and is more likely to affect older people living in communities [12].

Seniors in the Community: Risk Evaluation for Eating and Nutrition (SCREEN) is an example of a nutritional risk screening questionnaire designed for seniors who live in the local community. This questionnaire has undergone many adaptations [13] and validations [14,15,16]. Many versions of the SCREEN questionnaire have been useful in assessing diet [13], but also in assessing hospitalization outcomes and mortality [17]. The SCREEN-14 questionnaire (previously referred to as SCREEN II) has the highest reliability [18].

Elderly people living in the community may have problems with food consumption and are therefore prone to nutritional hazards [19,20]. This may lead to an increased nutritional risk and, consequently, deterioration of health and higher mortality [14,21,22]. According to a worldwide study using the SCREEN II questionnaire, the problem of high dietary risk among seniors (age 65 and older) affects 61.5% to 70.1% [23], and such risk is determined by individual and environmental factors.

Current data on the relationship between the nutritional risk of Polish seniors living in local communities and lifestyle indicate a significant relationship between this risk and alcohol consumption, smoking, moderate or low physical activity, too short (less than 6 h) or too long (more than 9 h) sleep time and living alone in a household [24].

Few studies indicate a relationship between the nutritional risk of older people living in the local environment and demographic characteristics, and these studies are not Polish. Those that have been published indicate more frequent occurrence of high nutritional risk among men [25,26], people in old age [27,28,29,30], rural inhabitants [31] and people living alone [26,27,28,29]. Studies that do not confirm these relationships are observed less frequently [32]. On the other hand, no studies assessing the relationship between the nutritional risk and socioeconomic status (SES) of elderly people living in the local environment have been observed.

From 2010 to 2030, the global population growth of people aged ≥65 years is expected to increase by 442 million, making it the fastest-growing age group [33]. Governments around the world have committed to promoting the independence and well-being of older people [34]. This policy is in line with the expectations of older people who want to stay in their own homes for as long as possible [35]. From the point of view of public health tasks, it would be important to systematically assess the nutritional risk of elderly people living in local communities, followed by specialized nutritional assistance for people at risk. An example of such aid is the Meals on Wheels (MOW) project implemented in the USA, which involves 1.5 million people living in various communities, both in rural and urban areas [36]. As part of the project, it was shown that older people awaiting help from the Ministry of National Defense project were more often widowed, less educated, of old age, had black skin and had symptoms of anxiety and depression. These seniors were more likely to be diagnosed with falls within the last month and more likely to fear falling than the national elderly population [37].

To sum up, in order to properly plan public health policy, it is necessary not only to assess the nutritional risk of older people living in local communities but also to identify population groups in which such risk occurs. Of particular importance in distinguishing these groups is the identification of demographic and socioeconomic characteristics determining high nutritional risk. Referring to the literature, it was hypothesized that gender, age, place of residence, region of residence as well as sociodemographic status influence the variation in dietary risk among older adults with different directions and strengths of association. In this context, the authors of this study assessed the relationship between the selected demographic characteristics (gender, age, place of residence and region of residence), socioeconomic status of polish elderly living in the community and different levels of nutritional risk.

## 2. Materials and Methods

### 2.1. Study Design and Sample

This paper is the result of a scientific study conducted under the title “Identification of nutritional risk in elderly people of different socioeconomic status and lifestyle”. The relationship between nutritional risk and selected lifestyle characteristics was presented in an earlier article published by the authors [24]. This article attempts to assess the relationship between dietary risk and selected demographic characteristics and declared socioeconomic status.

The research was carried out in two culturally and economically diverse regions in Poland. For example, in 2020, the Swietokrzyskie region was the region with the lowest GDP (71.9% of medium GDP per capita), while the Dolnonośląskie region was characterized by high GDP (110.7% of medium GDP per capita) [38]. The study was conducted from May 2021 to July 2021 in people aged 60 years and older. The sample for the study was arbitrarily selected by asking all associations, foundations and other senior citizen organizations from the Świętokrzyskie Voivodeship (City of Kielce, Kielce County and Sandomierski County) and in the Dolnośląskie Voivodeship (City of Wroclaw, Oława County) provinces for permission to participate in the study. Additionally, the snowball procedure was used to differentiate the sample. In total, 900 questionnaires were distributed to 21 organizations involved in senior activism. These organizations were willing to participate in the study. Inclusion criteria were age 60 and over and living in a local community. All respondents who declared their willingness to participate in the study were asked to provide questionnaires for the elderly people around their residence to fill out. In total, 466 surveys were collected, with 49 surveys eliminated due to non-response. The study included 417 people, 230 from the Świętokrzyskie Voivodeship and 187 from the Dolnośląskie Voivodeship. There were 312 women between 60 and 95 years old (70.5 ± 6.8 years) and 105 men between 60 and 90 years old (71.6 ± 6.3 years).

The study was performed following the Declaration of Helsinki [39]. The respondents gave their consent to participate in the study. Based on the provisions of the General Regulation of the European Parliament on Personal Data Protection, the personal data of the respondents were secured (GDPR 679/2016).

### 2.2. Questionnaire

The SCREEN-14 questionnaire, developed by Professor Heather Keller and colleagues at the University of Waterloo (Canada), was used to assess nutritional risk [11]. This questionnaire is the result of the development over the period 1999–2020 of earlier versions of screening questionnaires to assess the nutritional risk of older people living in communities in Canada (SCREEN I, SCREEN II) [11]. In addition, all versions of the SCREEN questionnaire have undergone many adaptations [13], validations [14,15,16] and nutrition studies [13]. Therefore, the final version of the SCREEN questionnaire, SCREEN-14, is a valid, useful and reliable tool that can identify older adults at nutrition risk [11,18]. The SCREEN-14 is a longer version of the earlier SCREEN questionnaires, allowing more risk factors to be identified, which can support the recognition of various areas for improvement and helps to match resources to specific needs [11]. The questionnaire includes 14 questions on weight change, weight gain, skipping meals; reducing food; subjective assessment of appetite; frequency of consumption of fruit and vegetables, meat and meat substitutes, milk and milk products; quantity of drinks; consumption of foods for special purposes; difficulties in biting, chewing and swallowing; circumstances in preparing and consuming food; and problems in buying food. Cronbach’s alpha coefficient was used to assess the reliability of the SCREEN-14 questionnaire used in the study. The Cronbach’s alpha coefficient for the variables of the present questionnaire was 0.726. All responses from the questionnaire were described by point indices according to the dietary risk assessment method [11]. For each answer, 0 to 4 points could be awarded. Less than 2 points for a particular response indicated a nutritional risk. After completing the SCREEN-14 questionnaire, a maximum of 64 points could be obtained. A cut-off point was taken for low and high nutritional risk, with 50 points or more for low nutritional risk and less than 50 points for high nutritional risk. The version of the SCREEN-14 questionnaire used in this study is provided in the Supplementary Materials (Questionnaire S1).

The demographic characterization of the treatment group was based on gender, age, place of residence and region of residence.

The following questions and point indices were used to assess SES:

1. Finantial situation was assessed on the basis of 2 questions. The first question was “How do you assess your financial situation?”—1 point was assigned for the “below average” response; 2 points for the answer “average”; 3 points for above average response. The second question was “How do you assess the financial situation of your household?”—1 point was assigned to the answer “it is not enough for me even for basic needs”; 2 points for the answer “I have to live very economically on a daily basis”; 3 points for the answer “it’s enough for me on a daily basis, but I have to save for larger purchases”; 4 points for the answer “is enough for me to do a lot without special saving” and 5 points for the answer “I can afford some luxuries”.
2. Education was assessed on the basis of the question “What is your education?”. Here, 1 point was assigned to the answer “primary education”; 2 points for the answer “vocational education”; 3 points for the answer “secondary education” and 4 points for the answer “higher education”.

Social activity was assessed on the basis of the question “Do you take an active part in different sociocultural encounters (e.g., in associations, circles, foundations, festivals and other senior citizen organizations)?”. Here, 1 point was assigned to the answer “no”; 2 points for the answer “yes, but rarely”; 3 points for the answer “yes, sometimes” and 4 points for the answer “yes, often”. Family relationships were assessed on the basis of the question “How do you rate your relationship with your immediate family?”, with the following answers: wrong (1 point); average (2 points); good (3 points); and very good (4 points). Here, 1 point was assigned to the answer “wrong”; 2 points for the answer “average”; 3 points for the answer “good” and 4 points for the answer “very good”. The SES of older people has been calculated using a method from the previously developed SES index [40,41]. The SES index was calculated for each participant by adding up the points for each variable (i.e., their material situation, education, social activity and family relationships). Cronbach’s alpha index was used to assess the integrity of the input data in the SES index [42]. Cronbach’s alpha for the variables of the SES index was 0.703. Based on the distribution of the SES tertiary index, groups of participants with low, medium and high SES indices were identified.

### 2.3. Statistical Analysis

The qualitative variables are presented in numerical and percentage (%) values. The chi-square test was used to check the differences between these variables.

Multivariate correspondence analysis (MCA) was used to determine the relationships between categories of variables describing nutritional risk (low and high), demographic characteristics (gender, age, place of residence and region of residence) and the size of the SES index (low, medium and high). A Burt matrix was used in the analysis, and the cumulative percentage of inertia and the scree criterion were taken as the criteria for choosing the number of dimensions of the projection space of the variables [42]. The diagram showed a clear break of the line at the second point, indicating the number of projection spaces. The percentage of inertia for the first and second dimensions was 19.93 and 15.13, respectively. On the scree diagram, a clear collapse of the straight line occurred at the second point, which indicates the number of projection space dimensions. The own value for the two dimensions was 0.45. Based on both criteria, a two-dimensional projection was used for graphic presentation. In order to interpret the obtained results, a hierarchical classification of variables was made using the Ward’s method, which estimates the distance between sets (clusters) of variables using the analysis of variance [41].

Logistic regression analysis was used to assess the relationship between the level of nutritional risk, demographic characteristics and the level of the SES index. A 95% confidence level was adopted for calculating the odds ratio (OR). Low dietary risk was chosen as the reference value in logistic regression analysis (OR = 1.00), using women for the variable gender, 60–74 for the age variable, rural area for where you live, Świętokrzyskie Voivodeship for the region of residence and low for the SES index. A *p* value < 0.05 was considered statistically significant for all tests.

The statistical analysis was carried out with STATISTICA software (version 13.3 PL, StatSoft Inc., Tulsa, OK, USA; StatSoft, Krakow, Poland) [43].

## 3. Results

### 3.1. Characteristics of Study Sample

Table 1 presents the demographic characteristics and SES expressed by the SES index levels of the study group. Less than 75% of the respondents were female and aged 60–74 years. Almost two thirds of the respondents came from towns with over 100,000 inhabitants and one third from the countryside. The Świętokrzyskie Voivodeship was represented in the study by over half of the respondents. Nearly 32% of respondents were characterized by low and nearly 25% by high socioeconomic status. The remaining percentage of respondents were characterized by medium socioeconomic status.

### 3.2. Nutritional Risk

**Figure 1:**
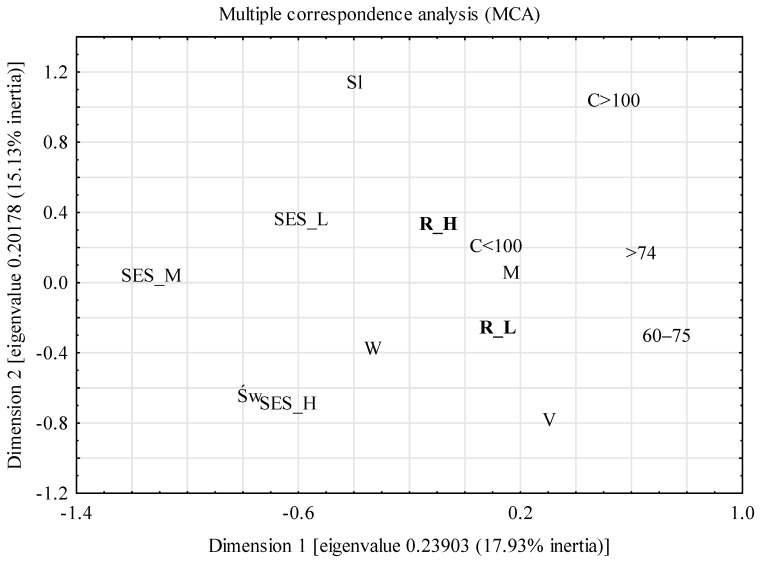
The structure of the relationship between the variables describing nutritional risk, selected demographic features and socioeconomic status. R_H—high nutritional risk; R_L—low nutritional risk; W—woman; M—man; 60–74—age in years; >75—age 75 and over; V—village; C < 100—city with up to 100,000 inhabitants; C > 100—city with over 100,000 inhabitants; Św—Świętokrzyskie Voivodeship; Sl—Dolnośląskie Voivodeship; SES_L—low socioeconomic status; SES_M—medium socioeconomic status; SES_H—high socioeconomic status.

**Figure 2:**
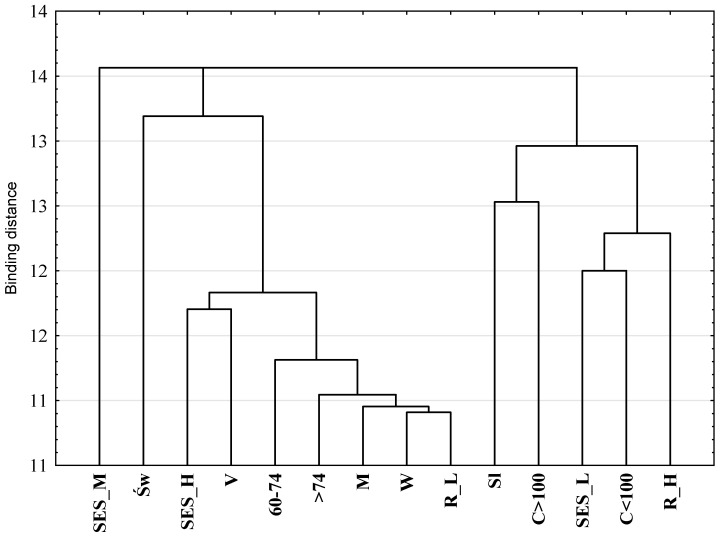
The hierarchical classification of variables describing nutritional risk, selected demographic features and declared socioeconomic status. R_H—high nutritional risk; R_L—low nutritional risk; W—woman; M—man; 60–74—age in years; >75—age 75 and over; C < 100—city with up to 100,000 inhabitants; V—village; C > 100—city with over 100,000 inhabitants; Św—Świętokrzyskie Voivodeship; Sl—Dolnośląskie Voivodeship; SES_L—low socioeconomic status; SES_M—medium socioeconomic status; SES_H—high socioeconomic status.

The structure of the relationship between the variables describing the level of nutritional risk, selected demographic features and the level of SES is presented in Figure 1. Using the hierarchical classification of Ward’s method, two sets of variables were selected (Figure 2). One set consisted of people with a high nutritional risk and, at the same time, characterized by such features as living in the Dolnośląskie Voivodeship, living in cities and low SES. The second set included people with a low nutritional risk, from the Świętokrzyskie Voivodeship, from rural areas and with a high SES. This set includes people of both sexes and all age categories.

Almost 80% of the respondents had a high nutritional risk. Gender and age were not variables significantly differentiating the level of nutritional risk of the subjects. Significantly more people at high nutritional risk came from the Dolnośląskie Voivodeship. On the other hand, in the Świętokrzyskie Voivodeship, the respondents were significantly more often characterized by a low nutritional risk. A significantly greater proportion of respondents from cities with a high nutritional risk was demonstrated in relation to a low nutritional risk. An inverse relationship was found in the case of respondents from rural areas. Low SES was significantly more often ascribed to people with high nutritional risk, and vice versa (Table 2).

The study showed that the inhabitants of the Dolnośląskie Voivodship were more than twice as likely to be at high nutritional risk when compared to inhabitants of the Świętokrzyskie Voivodship. Almost 2.5 times more often, people with such a nutritional risk lived in small towns (<100,000 inhabitants) and almost 1.25 times more often in large cities (>100,000 inhabitants) than rural regions. Additionally, residents of small towns were nearly half as likely to be exposed to high nutritional risk compared to residents of large towns. High nutritional risk was more than one-third more often observed among respondents with low socioeconomic status compared to respondents with high socioeconomic status (Table 3).

## 4. Discussion

The main aim of this work was to assess the relationship between the demographic characteristics (age, gender and place of residence) and socioeconomic status of older people in the community and nutritional risk. In this work, the authors placed an emphasis on examining the question of which of the listed socioeconomic factors determines the risk of malnutrition to the greatest extent—namely, that low SES was associated with a significantly higher nutritional risk. At the same time, age and sex were not factors significantly influencing differences in the level of nutritional risk. The study of nutritional risk is not a general field of research. Polish studies make it possible to compare dietary risks and conditions with the few studies carried out in other countries. It is, therefore, necessary to analyze the nutritional risk of elderly people living in the community, taking into account demographic characteristics and socioeconomic status.

Our study showed that nearly 80% of the respondents had a high nutritional risk. Research by Borkent et al. [23] showed that 61.5–70.1% of elderly people were at high nutritional risk. The differences may result from the fact that our study covered two voivodeships in Poland, while the study by Borkent et al. covered three countries: Canada, New Zealand and the Netherlands. Additionally, in our study, 75% of the respondents were aged 60–74, while in the second study, 65.8% of them were seniors aged 65–74. Different data were obtained by Morais et al., who showed a high nutritional risk only among 25.4% of the elderly [32]. It should also be noted that various questionnaires can be used to assess the level of nutritional risk. Therefore, comparing the research results of different authors may not be reliable.

In this study, a high nutritional risk was shown among 24.7% of people aged over 75. In Canada, this percentage was 24%, and it increased significantly with increasing age (up to 39% in the 79-year-old age group) [30,44]. Our research showed, however, that both the age and gender of the respondents did not significantly affect the level of nutritional risk. Locher et al. proved that high nutritional risk is more common in men, especially those who live alone [26]. It is mainly related to the purchase of low-quality food by single people and the inability or lack of motivation to prepare wholesome meals. In addition, eating meals together with the family contributes to the greater consumption of food products; thus, the nutritional risk is reduced [45]. In turn, Morais et al. showed that nutritional risk affects women (49.5%) and men (50.5%) to the same extent [32]. A weakness of our work may be the gender non-uniformity of the respondents, as women predominated (approximately 75%). In the first Polish study (PolSenior, *n* = 3751) on the analysis of health factors associated with poor nutritional status (PNS), malnutrition was found among 44.2% of respondents. Female gender (OR 1.72 (1.45–2.04)95% CI (Confidence Interval)), advanced age (OR 2.16 (1.80–2.58)), symptoms of depression (OR 11.52 (9.24–14.38)), cognitive impairment (OR 1.52 (1.20–1.93)], multi-morbidity (OR 1.27 (1.04–1.57)), anemia (OR 1.80 (1.41–2.29)) and complete edentulousness (OR 1.26 (1.06–1.49)) were independently correlated with PNS [46]. In the VARISAUDE study from Spain, which included 749 people over 65 living independently, malnutrition/risk of malnutrition was found in 14.3% of elderly people. In the group of women, polypharmacy and poor self-esteem were the most strongly correlated with the improper nutritional status. Among men, a positive effect on malnutrition/risk of malnutrition, obesity or overweight, depression and multidrug use was noted [47].

Wham et al. found that the older the age was, the higher the nutritional risk. The nutritional risk among people aged 75–79 years was 76.6%, while in those aged 80–85, it was around 10% higher [29]. The differences between the results of our own research and those of Wham et al. may result from differences in the age of respondents (60–95 years and 75–85 years, respectively).

The level of nutritional risk depends on the place of residence of the respondents and is significantly higher among people living in cites compared to people living in rural areas. At the same time, it has been shown that the nutritional risk is significantly higher in cities with fewer than 100,000 inhabitants than in cities with more than 100,000 inhabitants. These results differ from the data obtained by other authors. Crichton et al. showed that rural residents are more often affected by malnutrition, which is associated with higher nutritional risk [31]. The availability of different types of services varies between rural and urban areas. Many smaller towns are far away from urban areas, so long-distance travel is often required to make major purchases or access healthcare. However, seniors living in the countryside can use the help of family members much more often (shopping and preparing meals) than seniors living in cities. Some areas with city rights do not have welfare programs allowing seniors to enjoy free meals or centers for the elderly. Moreover, on the borders between town and village areas, grocery stores, pharmacies and access to public transport can be significantly limited [48]. The higher nutritional risk of city dwellers may also result from the fact that they have easier access to fast food restaurants, where they can eat relatively cheap meals and do not have to prepare them at home. However, this type of food is of low nutritional value and exposes seniors to the risk of nutritional deficiencies [49].

Based on this study, it was shown that low SES was associated with a significantly higher nutritional risk. The overall prevalence of malnutrition among elderly people with low SES around the world ranges from 28.9% to 48%. However, the lack of previous studies on the link between nutritional risk and the SES of elderly people, especially those living in Poland, significantly hinders the unambiguous interpretation of the results obtained. It has only been shown that low SES among the elderly is associated with poor nutritional status and, above all, with the lack of properly balanced food rations. On the other hand, risk factors for poor diet quality include financial and functional limitations, gender, smoking, place of residence and oral health [50].

Achieving the highest possible SES status in many studies is one of the basic factors for good health. Public health programs to promote healthy longevity should start much earlier than old age and target the poorer sections of society at all ages [51]. The design of future interventions to support older community-dwelling adults requires a clear understanding of the personal and contextual influences that affect patterns of food choice and consumption, including consideration of the importance of social and psychological factors. In addition, there are opportunities to intervene earlier in the life course—for example, including nutrient-dense foods. The most effective preventive efforts to promote good nutrition for healthier ageing may need to start ahead of age-related changes in physiology and function, including younger adulthood and at the retirement transition [52,53].

### Strengths and Limitations

Due to the fact that there is no terminological consensus between “nutritional risk” and “malnutrition risk” [10], the text of this paper uses the original terms used by the authors of the cited publications.

The strength of our research lies in the use of the SCREEN-14 questionnaire, which is widely used in different countries, to assess the nutritional risk of older people living in the community. The main advantage of this questionnaire is that it is possible to identify factors influencing a high nutritional risk at an early stage. Such measures can help to plan health policy properly. In addition, it is one of the few studies that identifies population groups (taking into account demographic characteristics and SES) that are at high nutritional risk.

It should be kept in mind that the present work is the result of a scientific activity, the results of which have already been partially published in an earlier paper [24]. In this situation, some of the terminological, methodological and statistical data may show features of similarity. 

The main limitation of the study is that it is a cross-sectional study. In the next step, the authors plan to carry out a longitudinal study to assess the nutritional risk, taking into account all Polish voivodeships. Only two voivodeships in Poland were selected for research here (Świętokrzyskie and Dolnośląskie). Consequently, the results may not be representative of seniors living nationwide. Additionally, women predominated among the study participants (nearly 75%). However, it should be emphasized that the sample size (417 people) was still powerful enough to detect statistically significant differences between the low- and high-risk food groups, taking into account the statistical analyses used.

The snowball method used in the selection of the research sample was aimed at differentiating the study population group in terms of social activity (activity in senior citizen organizations and lack thereof). However, despite the stated purpose of this method, it should be kept in mind that this method may tend to obtain a homogeneous sample in terms of environmental and social characteristics.

## 5. Conclusions

The level of nutritional risk among seniors is difficult to clearly define. They can be influenced by a number of factors (social, environmental and physical) that are closely related to each other. Despite the use of standardized assessment tools, they may not give a complete picture. Based on this study, nutritional risk factors were identified among Polish seniors, living in two voivodeships—Świętokrzyskie and Dolnośląskie. It has been shown that living in larger cities and low SES are closely related to a higher nutritional risk. At the same time, age and sex were not factors significantly influencing the level of nutritional risk. More extensive research is needed, taking into account different geographic locations. Identifying factors that increase nutritional risk among seniors can help in planning strategies to prevent nutritional and health problems in this age group.

## Figures and Tables

**Table 1:** Study sample characteristics.

Variables	*n* = 417	%
Gender		
Female	312	74.8
Male	105	25.2
Age		
60–74 years	314	75.3
75 years or more	103	24.7
Place of residence		
Rural area	122	29.3
City < 100,000 residents	35	8.4
City > 100,000 residents	260	62.3
Region of residence		
Świętokrzyskie Voivodeship	230	55.2
Silesia Voivodeship	187	44.8
Index SES		
Low	133	31.9
Medium	181	43.4
High	103	24.2

**Table 2:** Occurrence of nutritional risk depending on selected demographic characteristics and socioeconomic status.

Variables	Generally		Nutritional Risk Level				*p*
			High		Low		
	*n*	%	*n*	%	*n*	%	
Total	417	100.0	323	77.5	94	22.5	
Gender							
Female	312	74.8	240	74.3	72	76.7	1.000
Male	105	25.2	83	25.7	22	23.3	0.907
Age							
60–74 years	314	75.3	243	75.2	71	75.5	1.000
75 years or more	103	24.7	80	24.8	23	24.5	0.872
Place of residence							
Rural area	122	29.3	87	26.9	35	37.2	0.023
City < 100,000 residents	35	8.4	30	9.3	5	5.3	0.037
City > 100,000 residents	260	62.3	206	63.8	54	57.5	<0.001
Region of residence							
Świętokrzyskie Voivodeship	230	55.2	166	51.4	64	68.1	0.009
Silesia Voivodeship	187	44.8	157	48.6	30	31.9	0.044
Index SES							
Low	133	31.9	111	34.4	22	23.4	0.011
Medium	181	43.4	137	42.4	44	46.8	1.000
High	103	24.2	75	23.2	28	29.8	0.045

*p*—significance level (chi-square test).

**Table 3:** Associations between nutritional risk level and demographic characteristics and SES index in the study sample (adjusted odds ratios with 95% confidence intervals).

Variables	Nutritional Risk Level (Ref. ^1^ Low Level)	
	High Level	
	OR ^2^	*p*
Gender		
Female	1.00	
Male	1.13 (0.66–1.94)	0.653
Male	1.00	
Female	0.88 (0.56–1.34)	0.672
Age		
60–74 years	1.00	
75 years or more	1.02 (0.58–1.75)	0.954
75 years or more	1.00	
60–74 years	0.98 (0.62–1.53)	0.951
Place of residence		
Rural area	1.00	
City < 100,000 residents	2.41 (1.86–4.75)	0.041
City > 100,000 residents	1.24 (1.07–1.59)	0.037
City < 100,000 residents	1.00	
City > 100,000 residents	0.82 (0.70–0.92)	0.044
Rural area	0.44 (0.28–0.66)	0.036
City > 100,000 residents	1.00	
Rural area	0.80 (0.66–0.92)	0.030
City < 100,000 residents	1.48 (1.20–1.88)	0.045
Region of residence		
Świętokrzyskie Voivodeship	1.00	
Dolnośląskie Voivodeship	2.02 (1.23–3.28)	0.005
Dolnośląskie Voivodeship	1.00	
Świętokrzyskie Voivodeship	0.50 (0.31–0.80)	0.005
Index SES		
Low	1.00	
Medium	0.93 (0.54–1.20)	0.055
High	0.73 (0.53–0.99)	0.049
Medium	1.00	
High	0.79 (0.56–1.08)	0.053
Low	1.10 (0.96–1.28)	0.052
High	1.00	
Low	1.35 (1.09–1.55)	0.042
Medium	1.27 (0.93–1.46)	0.055

^1^ Reference value; ^2^ Odds Ratio; *p*—significance level of Wald’s test.

## Data Availability

Data presented in this study are available on request from the corresponding author.

## Supplementary Materials

The following supporting information can be downloaded at: https://www.mdpi.com/article/10.3390/nu14081582/s1, Questionnaire S1: SCREEN-14.
