# Supplementary material for: Differentiation of the Nutritional Risk of Polish Elderly People According to Selected Demographic Characteristics and Declared Socioeconomic Status
Source: Nutrients. 2022 Apr 11;14(8):1582. doi: 10.3390/nu14081582 (PMC9027789; doi:10.3390/nu14081582)
Supplement: Supplementary file 1 [file nutrients-14-01582-s001.zip › nutrients-1631104-supplementary.pdf]

Supplementary Materials:

Questionnaire S1: SCREEN-14

1a. Has your weight changed in the past 6 months?

1__Yes, I gained more than 4.5 kilograms.
2__Yes, I gained 2.5 to 4.5 kilograms.
3__Yes, increased but not more than 2.5 kilograms.
4__No, my weight stayed within a few pounds.
5__Yes, decreased but not by more than 2.5 kilograms
6__Yes, I lost 2.5 to 4.5 kilograms.
7__Yes, I lost more than 4.5 kilograms.
8__I don’t know how much I weigh or if my weight has changed.

1b. Have you been trying to change your weight in the past 6 months?

1__Yes.
2__No.
3__No, but it changed anyway.

1c. Do you think your weight is …?

1__More than it should be.
2__Just right.
3__Less than it should be

2. Do you skip meals?

1__Never or rarely.
2__Sometimes.
3__Often.
4__Almost every day.

3. Do you limit or avoid certain foods?

1__I eat most foods.
2__I limit some foods and I am managing fine.
3__I limit some foods and I am finding it difficult to manage.

4. How would you describe your appetite?

1__Very good.
2__Good.
3__Fair.
4__Poor.

5. How many pieces or servings of vegetables and fruit do you eat in a day? Vegetables and fruit can be canned, fresh, or frozen.

1__Five or more.
2__Four.
3__Three.
4__Two.
5__Less than two.

6. How often do you eat meat, eggs, fish, poultry, tofu, dried peas, beans, lentils, nuts, or nut butters?

1__Two or more times a day.
2__One to two times a day.
3__Once a day.
4__Less than once a day.

7. How often do you have milk, soy beverages, or milk products such as cheese, yogurt, or kefir?

1__Three or more times a day.
2__Two to three times a day.
3__One to two times a day.
4__Usually once a day.
5__Less than once a day.

8. How much fluid do you drink in a day? Examples are water, tea, coffee, herbal drinks, juice, and soft drinks, but NOT alcohol.

1__Eight or more cups.
2__Five to seven cups.
3__Three to four cups.
4__About two cups.
5__Less than two cups.

9. Do you cough, choke or have pain when swallowing food OR fluids?

1__Never.
2__Rarely.
3__Sometimes.
4__Often or always.

10. Is biting or chewing food difficult for you?

1__Never.
2__Rarely.
3__Sometimes.
4__Often or always.

11. Do you use commercial meal replacements or supplements? Examples are shakes, puddings, or energy bars.

1__Never or rarely.
2__Sometimes.
3__Often or always.

12. Do you eat one or more meals a day with someone?

1__Never or rarely.
2__Sometimes.
3__Often.
4__Almost always.

13a. Who usually prepares your meals?

1__I do.
2__I share my cooking with someone else.
3__Someone else cooks most of my meals.

13b. Which statement best describes meal preparation for you?

1__I enjoy cooking most of my meals.
2__I sometimes find cooking a chore.
3__I usually find cooking a chore.
4__I'm satisfied with the quality of food prepared by others.
5__I'm not satisfied with the quality of food prepared by others.

14. Do you have any problems getting your groceries? Problems can be poor health or disability, limited income, lack of transportation, weather conditions, or finding someone to shop.

1__Never or rarely.
2__Sometimes.
3__Often.
4__Always.
